# Differential Diagnosis of Intrauterine Pregnancy Based on Clinical Observation1Read before the Section of Obstetrics of the Buffalo Academy of Medicine, April 25, 1911.

**Published:** 1911-06

**Authors:** Ludwik Schroeter

**Affiliations:** Buffalo, N. Y.; Attending Obstetrician, St. Mary’s Maternity Hospital; Assistant Attending Obstetrician, Buffalo General Hospital; Clinical Instructor in Obstetrics, Medical Department, University of Buffalo


### Differential Diagnosis of Intrauterine Pregnancy Based on Clinical Observation[1]

By LUDWIK SCHROETER, M.D. (Bern)
Buffalo, N. Y.

Attending Obstetrician, St. Mary's Maternity Hospital
Assistant Attending Obstetrician, Buffalo General Hospital
Clinical Instructor in Obstetrics, Medical Department, University of Buffalo

I MUST admit that I hesitated quite a while before I decided upon the subject of this paper. Not having a single new symptom to add and only very few original observations to offer, it seems presumptuous on my part to discuss such a trite subject before this section. Having, however, had on several occasions the mortification of both inability to make and of mistakes in the diagnosis of pregnancy, and being satisfied that I am not an exception in this respect, I thought it apropos to review this old field of elementary obstetrics.

You will admit that in the great majority of cases it is the patient who makes the diagnosis of her pregnancy. With the exception of two classes of women—the unhappy bearers of undesirable fruit and the poor fruitless desirers of progeny—we generally can rely upon the diagnosis of our patients when the question of pregnancy is concerned. Nevertheless, it may bring the practitioner into an unenviable position if he omits to corroborate such a diagnosis by a thorough objective examination. It seems, however, to be the practice of some physicians to take the statements of their patients for granted.

Some fifteen years ago I was called to attend a woman in labor. She was a multipara about forty years old. The family physician who had been engaged for the case could not be located, so I was supposed to substitute for him. Upon entering the lying-in room I found everything prepared for the expected newcomer. The patient told me that she had been having labor pains for the last few hours. She begged me "to help her." While I was preparing to make an external examination I noticed that there was no tumor visible and aside from a slight bloating of the abdomen no suggestion even of an advanced pregnancy. I asked the patient on what ground she suspected that she was going to give birth to a child. She was highly indignant at this question. She told me that, having given birth to five children, she had sufficient experience; that nine months had elapsed since her last menstruation, and that she had felt life since her fifth month of pregnancy, and so on. She insisted again that I should help her. An internal examination revealed that she not only was not in labor, but not even pregnant.

---

1. Read before the Section of Obstetrics of the Buffalo Academy of Medicine, April 25, 1911.

I broke the news as gently as I could to the family, upon which I was promptly discharged from the case. On leaving the room I overheard some uncomplimentary remarks about the inexperienced doctor and the request that the family physician be sent for immediately. I wished then that I could have seen the expression of the doctor when he faced the situation.

The experience with this case saved me mortification in another instance several years later. A man called at my office to engage me for the confinement of his wife who, he said, was seven months pregnant with her first child. I explained to him that I must see the patient and examine her. He replied that his wife is very modest and oversensitive, and absolutely refuses to be examined until labor sets in. I flatly refused to take the case under such condition. A few days later I was called to see the patient. She gave me a history of having missed her changes for three months, that later she did menstruate twice, but very scantily. She had been suffering with morning vomiting for several months and had experienced quickening for the last two months. On examination it appeared to be a pure and simple imaginary pregnancy.

Of the many subjective symptoms of gravidity only one is of real and essential importance—the suppression of menstruation; but while it leads to the proper diagnosis in the great majority of cases it cannot always be fully relied upon. First there are women with atypical menstruation—every two or even every six months. In some women the menstruation may appear once, sometimes twice after conception, although in a very scanty amount. Then there are women who bear children without ever having menstruated. The menstruation may be suppressed during lactation, in chlorosis, tuberculosis, syphilis and other wasting diseases. Early menopause as a cause of amenorrhea, as was the first case reported by me tonight, must be constantly borne in mind. Last, but not least, intentional misrepresentation by the patient may mislead and sometimes misguide the physician into interference so much desired by such women.

An objective examination makes the diagnosis of an early pregnancy very probable for any one accustomed to bimanual examination. Care should be taken, however, that the bladder be empty. My invariable custom is to catheterise the patient in every instance in which I encounter any difficulty in palpating the uterus. The increase in size alone does not prove much. A chronic metritis, a fibroid tumor, a subinvoluted uterus of a nursing woman or a hematometrium may and often does mislead the beginner. If the increase in size is in the antero-posterior diameter chiefly, it speaks more in favor of pregnancy than of any other condition. The consistency of the uterus, its doughy feeling "in toto" or in parts only, especially in the region of the utero tubal angles, is of more importance and strongly indicates pregnancy. In many cases the uterus is so soft that at first on bimanual examination the uterine body may not be felt, and an inexperienced man may consider the cervix, if elongated, as the whole uterus and exclude pregnancy on the presumption of the small size and normal consistency of the supposed uterus.

The symptom generally accepted as very reliable—the softening of the cervix—is sometimes misleading. Softness of the cervix exists shortly before and during menstruation; in women suffering for a long time from leucorrhea and is also frequently a concomitant of uterine tumors. In many cases I have found a soft cervix in non-gravid women without any apparent cause. Moreover, in some exceptional cases the cervix may remain hard during the full term of pregnancy.

With the advance of the pregnant state, about the middle of the third month, the doughy feeling gives place to a cystic sensation, which added to other possible and probable signs brings the diagnosis of pregnancy almost close to a certainty. Hegar's sign is based on these changes in the consistency of the uterine wall. Having noticed on repeated examinations that the softest parts are the lower uterine and upper cervical, Hegar found that on bimanual palpation this portion of the uterus is not felt at all, that at this point the examining fingers of both hands touch. We have, then, the impression as if the uterine body is separated from the vaginal portion. In difficult cases Hegar advises to feel for his sign by rectal examination. In examining a supposed pregnant woman during the early months, I am always on the lookout for this sign and when present I rely on it more than on all other probable and possible signs of pregnancy combined.

Objective symptoms other than those found on bimanual examination as pigmentation of the linea alba and the nipples, the characteristic discoloration of the vestibulum vagina and uterine os, the presence of colostrum in the breasts are unreliable, as they may be found in conditions other than pregnancy and also in non-pregnant multiparæ. Sometimes we may find great difficulty on bimanual examination. A retroflexed uterus may be so enlarged and softened on account of passive congestion that it readily impresses the examiner as an early pregnancy. In such cases it is imperative to replace the uterus whenever possible before we can make a diagnosis.

I know of two cases of considerable difficulty in differentiating cystic fibromyomata of the uterus from pregnancy. In one the tumor felt and looked so much like a four months' pregnant uterus that after having opened the abdomen the surgeon still hesitated—the opinion of several experienced men present being in favor of a gravid uterus—and only after repeated aspirations were negative did he decide to proceed with the operation.

The differential diagnosis between intra- and extrauterine pregnancy during the first few months is sometimes impossible. Great difficulty may confront us in cases of missed abortion. I know of several patients whose conditions under similar circumstances have been mistaken by different men of considerable experience. At all events in the early months the negative diagnosis of pregnancy is more difficult than the positive. While mistakes are more frequent during the first few months of pregnancy, the danger of such error is much greater during the second half. With the positive signs—fetal heart sounds, feeling of the fetal parts, hearing and feeling of the fetal movements and the sensation of ballottement,—it seems that a mistaken diagnosis would be hardly possible. Still these mistakes do occur and in some very exceptional cases seem to be unavoidable.

Last year I hesitated with the diagnosis in two cases—one six and the other five months pregnant. The first carried a dead fetus and had a tumor irregular in outline and of indefinite consistency. The other woman had been bleeding quite profusely every few weeks. No positive sign was present. The configuration of the uterus was very irregular and it was of hard consistency. She delivered herself at term of a living child.

I know of two cases operated on by a surgeon for fibromyomata which turned out to be pregnancies of the second half. Another surgeon discovered his mistake only after he found a foot of a five months' fetus in the instrument which was supposed to remove a uterine tumor.

The diagnosis may be very difficult in those exceptional cases of hydatid moles, when the uterus during the third month reaches the size of a six or seven months pregnancy and where there are intercurrent hemorrhages. The most experienced and most careful observer will be confused in similar cases. While an ovarian cyst, particularly if multilocular, may puzzle the examining physician, there is hardly an excuse for mistaking a very extensive ascites for pregnancy or vice versa.

A case is reported in literature where an overdistended bladder containing a large stone led to a mistake in the diagnosis, the mobility of the stone having given the examiner the sensation of ballottement. Multiple pregnancies have occasionally misled practitioners and been mistaken for tumors. But one of the most difficult diagnoses, where many errors are being made, is a case of excessive hydramnion, the more so because such conditions are quite frequently complicated by a multiplicity of fetuses.

Well do I remember the first case of this kind. A woman entered the medical ward of a Warsaw Hospital in Poland. She stayed there over a month and was treated for Bright's disease. Pronounced edema of the lower extremities, urinary findings and what was supposed to be an extensive ascites led to the diagnosis. The patient, a multipara, did not consider herself pregnant and was supposed to be past her menopause. The chief of the clinic, on returning from his vacation, when shown the patient for the first time, after a cursory percussion and auscultation, made a vaginal examination and ordered the patient to be transferred to the maternity to the great astonishment of his assistants. The director of the obstetrical ward, after a careful examination only shook his head doubtfully and ordered the patient to be kept under observation. A week or so later the woman's condition became serious on account of impaired respiration. The obstetrician then, only after another examination, decided to act on the advice of his internist friend. He introduced bougies into the uterus and next day the patient corroborated the wonderfully exact diagnosis of Professor Lambl. She delivered herself of a pair of twins, preceded and followed by an immense amount of amniotic fluid.

The second case of this kind did not terminate so successfully. A woman of over forty years missed her menstruation twice. She thought herself pregnant. But a few weeks after she missed her changes twice she started to flow at short intervals for about two months. She harbored an immense intra-abdominal tumor of irregular form; distinct fluctuation could be felt. Several physicians of our city did see and examine this patient and every one diagnosed an ovarian cyst. The last man who saw the case took into consideration the possibility of pregnancy and measured the uterine cavity; the sound showed only three inches so he excluded the possibility of gravidity and decided to operate for an ovarian cyst. The trocar, however, proved the diagnosis to be wrong—a very large amount of amniotic fluid escaped. Porro operation showed the presence of twins. The woman succumbed a week or so later from general sepsis.

The reverse mistake—to consider a tumor a pregnancy is of much rarer occurrence. I remember two such cases. I was sent for by a midwife to help her in an obstetrical case. She spent the entire day with the patient who was a multipara. "Notwithstanding constant pains there was no progress" was the midwife's statement. On examination I found the abdomen enlarged by a tumor corresponding to a six months' pregnancy, of unequal consistency, rather cystic; no fetal heart could be heard, no parts felt, no intermittent contraction of the uterus during a prolonged examination. On vaginal examination it was quite difficult to locate the cervix; at last I found it high over the symphysis, of firm consistency and peculiarly flattened; the uterine os was closed—the midwife was positive that there was full dilatation. Through the culdesac no fetal parts could be felt. I told the family that the woman was not in labor, probably not pregnant, but I failed otherwise to make a diagnosis of the tumor. As a consequence two other physicians were called to see the patient on the next day. The diagnosis of sarcoma was made after a prolonged examination under chloroform. Incidentally, I will state that some six months later I happened to meet the woman at the house of another patient. She certainly had her laugh. She told me that several weeks after that eventful night "something gave away" and that for hours she had been discharging through the vagina a very foul smelling fluid.

A somewhat similar case occurred in my practice last year. A young Italian woman showed a doughy feeling tumor reaching two fingers above the umbilicus. The tumor looked and felt more like a six months' pregnant uterus than anything else. No mistake could be made, however, in this case as it was three months after her labor. Dr. James E. King operated on her at the Buffalo General Hospital by opening the posterior culdesac and emptied a large amount of pus.

In another instance an unmarried woman, twenty-five years old, who two years before gave birth to a full term child missed her changes for over two months, then had been flowing at irregular intervals. She had an abdominal tumor corresponding to a four months' pregnancy. I considered her pregnant even though she assured me that this was impossible. I kept her under observation for nearly four months. The tumor continued to increase in size to that of a gravid uterus, but her menstruation returned and was, according to the patient's statement, regular. No positive signs of pregnancy being present I decided to explore the uterine cavity. After having dilated the cervix by Hegar's bougies I could feel the uterus to be empty. A succeeding operation proved a multiocular ovarian cyst.

The difficulty of the diagnosis may be extraordinary if pregnancy takes place in a fibromatous or carcinomatous uterus, or where there is a combination of pregnancy with tumors, or a combination of an intrauterine with an extrauterine pregnancy. In any very complicated case a deep narcosis may be required. A combined rectal and vaginal examination, while the cervix is being pulled down with a tenaculum, may be necessary. It is possible that in doubtful cases where the most careful examinations are of no avail Radiology may clear up a diagnosis.

Whatever the complication is it should be a rule for every physician always to think of the possibility of pregnancy when there is the least likelihood of it and under no circumstances to decide on any operative intervention until pregnancy is positively excluded. A single examination will not be sufficient in a number of cases; it may be necessary to examine the patient on several occasions.

It is of the greatest importance for the physician's reputation as well as for our patient's welfare, both physical and moral, that one should not commit himself in making or excluding a diagnosis of pregnancy unless one is absolutely certain of his findings. Any mistake may be forgiven and forgotten by the public, but a wrong diagnosis of pregnancy, whether positive or negative, will certainly remain the topic of conversation and comment for a generation.

It would indeed be both interesting and instructive to our profession if our mistakes of this character should be reported and discussed at our meetings. Such a practice would save the younger and some of the older men also from rash diagnoses and their patients from unnecessary mutilating operations. Let this consideration be my excuse for reading this paper.

415 Franklin Street.

